# Technical Validation of a Training Workstation for Magnet-Based Ultrasound Guidance of Fine-Needle Punctures

**DOI:** 10.3390/s25134102

**Published:** 2025-06-30

**Authors:** Christian Kühnel, Martin Freesmeyer, Falk Gühne, Leonie Schreiber, Steffen Schrott, Reno Popp, Philipp Seifert

**Affiliations:** 1 Clinic of Nuclear Medicine, Jena University Hospital, Am Klinikum 1, 07747 Jena, Germany; christian.kuehnel@med.uni-jena.de (C.K.);; 2 eZono AG, 07743 Jena, Germany

**Keywords:** training workstation, ultrasound guided, needle puncture, augmented reality

## Abstract

It has been demonstrated that needle guidance systems can enhance the precision and safety of ultrasound-guided punctures in human medicine. Systems that permit the utilization of commercially available standard needles, instead of those that necessitate the acquisition of costly, proprietary needles, are of particular interest. The objective of this phantom study is to evaluate the reliability and accuracy of magnet-based ultrasound needle guidance systems, which superimpose the position of the needle tip and a predictive trajectory line on the live ultrasound image. We conducted fine-needle aspiration cytology of thyroid nodules. The needles utilized in these procedures are of a slender gauge (21–27G), with lengths ranging from 40 to 80 mm. A dedicated training workstation with integrated software-based analyses of the movement of the needle tip was utilized in 240 standardized phantom punctures (angle: 45°; target depth: 20 mm). No system failures occurred, and the target achieved its aim in all cases. The analysis of the software revealed stable procedural parameters with minor relative deviations from the predefined reference values regarding the distance of needle tip movement (−4.2% to +6.7%), needle tilt (−6.4% to +9.6%), and penetration depth (−7.5% to +4.5%). These deviations appeared to increase with the use of thin needles and, to a lesser extent, long needles. They are attributed to the slight bending of the needle inside the (phantom) tissue. The training workstation we employed is thus suitable for use in educational settings. Nevertheless, in intricate clinical puncture scenarios—for instance, in the case of unfavorable localized small lesions near critical anatomical structures, particularly those involving thin needles—caution is advised, and the system should not be relied upon exclusively.

## 1. Introduction

Training individuals in how to use ultrasound-guided puncture techniques is particularly crucial for clinical practice, as such training enables healthcare professionals to develop the necessary skills to perform these procedures safely and effectively. Ultrasound-guided punctures, as minimally invasive procedures, address a wide range of medical issues [1,2]. With sufficient training and practice, high levels of accuracy can be achieved while maintaining a reasonable degree of safety.

Various studies have revealed the effectiveness of ultrasound training workstations and virtual simulation systems in improving medical education and procedural accuracy [3]. For example, Ebner et al. demonstrated that the application of an augmented reality ultrasound trainer significantly improves kidney measurement accuracy for medical students [4]. Additionally, Kuok et al. reported superior ultrasound imaging skills in pediatricians after they underwent simulation-based ultrasound training courses [5]. Furthermore, Fulton et al. demonstrated that simulation-based training significantly enhances the performance of ultrasound-guided biopsies by reducing the procedural time, the number of skin punctures, and the number of needle adjustments [6]. Advanced virtual trainers and simulation platforms, such as the Perk Tutor, have been developed to train clinicians in image-guided interventions [7]. For instance, Law et al. described an approach of using an accelerometer to achieve high precision in the performance of ultrasound-guided percutaneous breast needle biopsies [8].

The use of advanced ultrasound needle guidance systems (US-NGSs) improves the accuracy of needle placement and reduces the rate of complications. The superior safety profile of ultrasound-guided biopsies ensures successful first attempts, thereby reducing complications such as injury and hematoma when the techniques are applied in clinical scenarios [9].

The puncture of thyroid nodules is of particular interest in this ex vivo phantom study. Due to the high prevalence of these lesions, especially in iodine-deficient areas such as Germany, fine-needle aspiration cytology is frequently performed in clinical routine [10]. Despite the thyroid’s relatively superficial location, target lesions can be unfavorably localized, e.g., in immediate proximity to the carotid artery. Due to the neck’s narrow anatomy with closely adjacent risk structures, it requires precise puncture techniques; however, complications still occur despite the use of thin needles [11].

Since 2016, we have used a US-NGS provided by eZono™ (eZGuide; eZono AG, Jena, Germany) for puncturing thyroid nodules in our clinic, and our experiences with this system have been positive [12]. Unlike most US-NGSs, which rely on costly, manufacturer-exclusive needles, the eZono™ system uses standard needles. This US-NGS is a versatile tool in clinical medicine and educational phantom training in general due to its ease of use, which is achieved through the simple magnetization of a wide range of commercially available standard needles [13,14,15,16]. In particular, Lee et al. demonstrated that the system can improve the accuracy of thyroid nodule punctures, especially for less-experienced operators [17].

However, the thin needles (21–27G) recommended for use in these interventions present technical challenges for the US-NGS [18,19]. For some thin needles, the ferromagnetic material may not be sufficient to ensure stable detection in the magnetic field. Metal objects (such as watches, cell phones, or cables) being near the NGS can then lead to its failure; this is particularly problematic when it occurs during puncture. Additionally, it is possible for the needle to bend within the tissue. Presently, there is a lack of data on the accuracy of needle position tracking with such thin needles, highlighting a critical gap in the current validation of this technology. Therefore, it is crucial to evaluate the reliability of the needle position within the augmented reality environment provided by the eZono™ system.

We intended to use the US-NGS both safely in clinical practice and as a training tool for inexperienced puncturists. To achieve this, it was necessary to evaluate the reliability of the system within a standardized experimental setup. Consequently, the aim of this study was to systematically evaluate the precision and safety of a magnet-based US-NGS for standard needles, particularly focusing on the use of thin standard needles in an ex vivo phantom study. We designed a training workstation that offers numerous advantages for the education and training of junior colleagues and students. This workstation proved to be equally suitable for the assessment of the reliability of the US-NGS under investigation.

## 2. Materials and Methods

In this study, we conducted an experimental ex vivo approach to validate the technical accuracy of the eZono™ 4000 US-NGS. Needles of different sizes and strengths were magnetized and positioned in a custom-built setup, designed to simulate clinical conditions in a reproducible setting.

The US-NGS is preconfigured for a variety of different needle sizes and manufacturers, though it is possible to add customized needles. For the purpose of this study, three needles of different strengths and lengths were selected, which are detailed below:

- Sterican 21G 40 mm (REF 4657527, B Braun SE, Melsungen, Germany);
- Sterican 27G 40 mm (REF 4657705, B Braun SE, Melsungen, Germany);
- Sterican 21G 80 mm (REF 4665465, B Braun SE, Melsungen, Germany).

A custom silicon phantom block was fabricated from plastisol with inorganic granular particles, and this block measured 130 × 85 × 40 mm (length × width × height). The plastisol was dotted with different particle levels to alter its echogenic characteristics, allowing it to be compatible with the different tissue types of human necks. The phantom contained a 6 mm diameter tubular target structure, positioned at a depth of 17–23 mm. The goal of all of the procedures was to reach the center of this target.

### 2.1. Training Workstation

The training workstation comprised the US-NGS, which was mounted to a mobile cart (Hefei Mt Medical Co., Ltd., Hefei, China) using a monitor bracket (ricoo, E.N.Z. Engineering GmbH, Kenzingen, Germany). A trainer observed the puncture procedures on a monitor positioned at the back of the cart (DELL, Frankfurt am Main, Germany) that was connected to a mini-PC (Intel, Santa Clara, CA, USA). The procedures were simultaneously recorded by three different systems to ensure comprehensive documentation and analysis.

**Figure 1:**
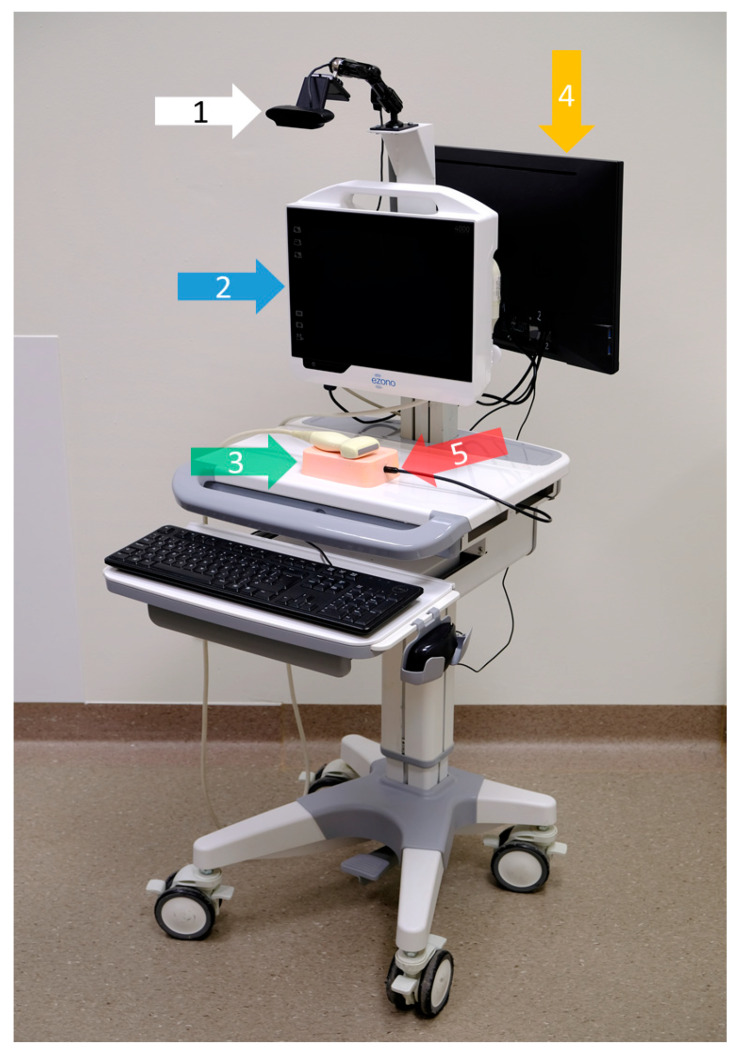
Training workstation on a mobile cart, viewed from the trainee’s side. The white arrow (1) points to the overhead observation camera. The blue arrow (2) shows the US-NGS device, which is equipped with a 12 MHz linear transducer. The green arrow (3) highlights the utilized plastisol phantom. The orange arrow (4) points to the trainer’s monitor on the back of the cart. The monitor is connected to a mini-PC installed in a drawer under the table to record the data and operate the analysis software. The red arrow (5) shows the endoscopic camera. The workstation is portable and uses only a single power supply; its height is also adjustable. Close-ups of the trainer’s monitor (4), as well as the views of the overhead observation camera (1) and the endoscopic camera (5), are shown in Figure 2.

Firstly, the images of the US-NGS were transmitted to the trainer’s monitor via a USB-to-LAN converter. Secondly, a camera (Logitech Europe S.A., Lausanne, Switzerland), positioned above the trainee’s side, captured the handling of the needle and ultrasound transducer from above. Thirdly, inside the phantom, a USB endoscope (USB2.0 PC Camera; Microsoft Corporation, Redmond, WA, USA) was embedded to provide the internal views of the puncture target (Figure 1).

**Figure 2:**
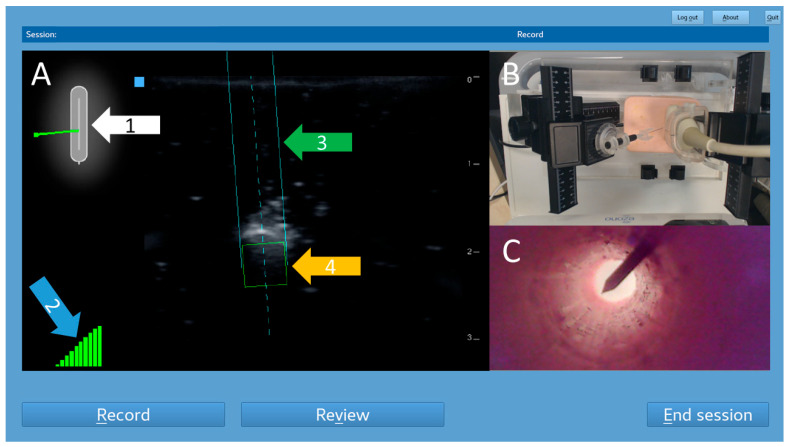
Screenshot of the trainer’s monitor. This example shows an out-of-plane puncture. The left part is a real-time mirror image of the US-NGS (**A**). The sensors inside the ultrasound transducer recognized the position of the previously magnetized needle. A 2D schematic overview shows the needle’s position in relation to the ultrasound probe (white arrow, 1). A 10-stage display of the current stability of the magnetic field was provided (blue arrow, 2). The respective location information was superimposed on the ultrasound images as trajectory lines (green arrow, 3) and a target box for the needle tip (orange arrow, 4) in real time. Video images captured by the observation camera above the trainee’s side were displayed in the top right corner of the trainer’s screen (**B**). Images from the endoscopic camera within the puncture target tube were displayed in the bottom right corner (**C**).

This setup allowed for real-time observations and recordings of all the fine-needle punctures to take place, providing the detailed options of the analysis. The integration of multiple cameras and the ability to live stream ensured that both the trainee and a potential trainer (not applicable for our data) were able to monitor and evaluate the quality of the performance effectively, facilitating an optimal learning environment. The data acquisition started with the movement of the first needle and ended at the moment when the needle tip was detected within the target (Figure 2).

### 2.2. Puncture Procedures

A single observer performed all the puncture procedures with a customized puncture application system (PAS). The aim was to position the needle tip inside the tubular target structure. The observer was only able to see the ultrasound images (including the superimposed needle guidance graphics), not the trainer’s monitor. Several parameters (injection type, number of readjustments, puncture time, distance between needle tip and target, needle tilt, distance of backward movement) were predefined (reference values) to enable comparability with the values recorded by the analysis software. Given that the system is to be utilized in the fields of education and training, the readjustment parameter is intended to specify the frequency with which a change has been made (ideally this would be “0”). The settings were selected with the objective of simulating the conditions of a clinical puncture. A timer was set to predefine the puncture time. An example of an out-of-plane puncture is shown in Supplementary Video S1.

Several different procedural data were recorded using the custom analysis software (created by eZono™) running on a mini-PC. The following performance, safety, and quality metrics were measured for each puncture, and they were provided in a dedicated procedure report:

- Injection type: Out-of-plane versus in-plane with or without readjustments.
- Puncture time [s]: the time between the insertion of the needle tip into the phantom surface and the needle tip’s penetration of the target.
- Total distance of needle tip movement [mm]: all forward and backward movements during the entire puncture procedure.
- Maximum penetration depth of needle tip [mm]: the deepest point of the penetration of the needle tip during the entire puncture procedure, perpendicular to the surface of the phantom.
- Needle tilt [degrees]: the average value of the angle between the needle and the phantom surface during the entire puncture procedure.
- Number of trajectory readjustments [n]: backward movements inside the phantom (given that the system is to be utilized in the fields of education and training, the readjustment parameter is intended to specify the frequency with which a change has been made; ideally, this would be 0).
- Total distance of reverse needle tip movement [mm]: only backward movements during the entire puncture procedure.
- Two-dimensional graphical plots of (a) the penetration depth of the needle tip (perpendicular to the surface of the phantom) over time and (b) the penetration depth of the needle tip (perpendicular to the surface of the phantom) over the horizontal needle tip position.
- All punctures were performed at a fixed angle of 45 degrees.

### 2.3. Puncture Application System (PAS)

**Figure 3:**
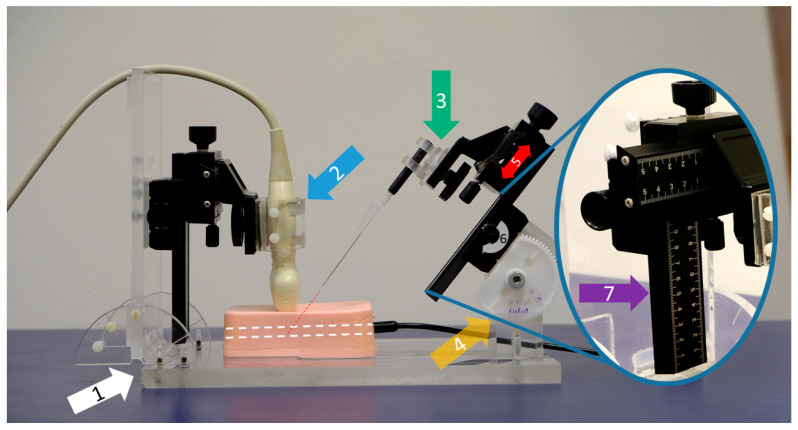
Customized puncture application system (PAS). This example shows an out-of-plane puncture using a 21G 40 mm needle. The system stands on a solid base plate (white arrow, 1). The ultrasound probe is fixed in a holding clamp (blue arrow, 2) and can be rotated for either in- or out-of-plane procedures. A large variety of different needles can be clamped into a flexible cone holder (green arrow, 3). The puncture angle is manually adjustable via a sturdy bicycle construction (yellow arrow, 4) in order to move the needle precisely along the red dotted line to ensure that it is pointing into the phantom’s target (white dotted line). In our study, a needle tilt of 45 degrees was set for all procedures. The needle carriage is characterized by its linear movability (red arrow, 5) via a side-mounted rotary knob (gray curved arrow, 6). The distances traveled can be read on the calibrated ruler (purple arrow, 7).

A PAS was developed to verify the accuracy of the data collected by the software. Distances and angles were freely configured. The PAS was made of polymethyl methacrylate (PMMA) and meticulously designed: it included two 4-axis macro cross slides (Baoblaze 4 Makro cross slide, Shenzhen Luzheng Technology Co., Shenzhen, China) mounted to a robust base plate, allowing for the precise configuration of the puncture settings. Distances and angles were measured using markers placed on the 4-axis macro slides (Figure 3).

One side of this construction was specifically engineered to accommodate the ultrasound transducer, allowing it to be securely attached and accurately angled. The macro cross slide mechanism ensured reproducible adjustments and precise movements, critical for consistent measurement outcomes. Depending on the type of measurement required, whether out-of-plane or in-plane techniques, the ultrasound transducer could be rotated by 90°, providing versatility in its application. The second macro cross slide was designed to facilitate the attachment of various needles to a cone holder, which offers multiple degrees of freedom, including rotation, swivel, and tilt. This design allows for complex and precise positioning of the needles and accurate and reliable measurements.

To minimize any potential interference with the magnetic sensors of the US-NGS, the mounting screws and hinges were crafted from PMMA or polyamide materials. These materials were selected specifically for their non-interfering properties, ensuring that the sensitive measurements obtained by the US-NGS were unaffected by external interference. This carefully designed setup provided a highly adaptable and interference-free environment for precise measurements to be taken.

### 2.4. Data Analyses and Statistics

Data were recorded using the Excel software (version 2016, Microsoft Corporation, Redmond, WA, USA) and transferred to the R programming language to ensure robust and reproducible calculation results. The mean ± standard deviation, median and range, and relative deviations were calculated. The ANOVA and Mann–Whitney U-tests were used for the analyses of potential differences between the groups (out-of-plane/in-plane, with/without readjustments, needle type). *p* < 0.05 was considered significant.

## 3. Results

**Figure 4:**
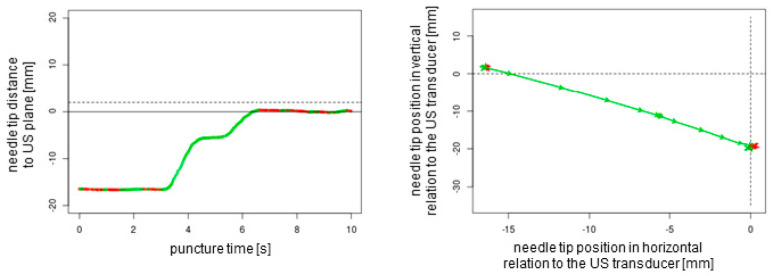
The two-dimensional graphical plots of the NGS data captured during an out-of-plane puncture without readjustment of the needle tip. Green lines show forward movements, and red lines display backward movements or standstill. The dotted lines represent the ultrasound transducer planes.

**Figure 5:**
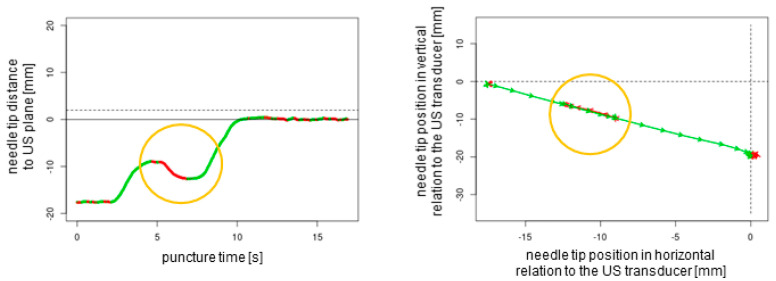
Out-of-plane puncture with readjustment of the needle tip (orange circles). The dotted lines represent the ultrasound transducer planes.

**Figure 6:**
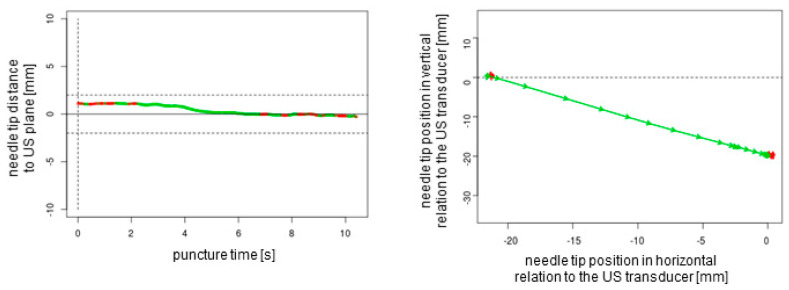
In-plane puncture without readjustment of the needle tip. The dotted lines represent the ultrasound transducer planes.

**Figure 7:**
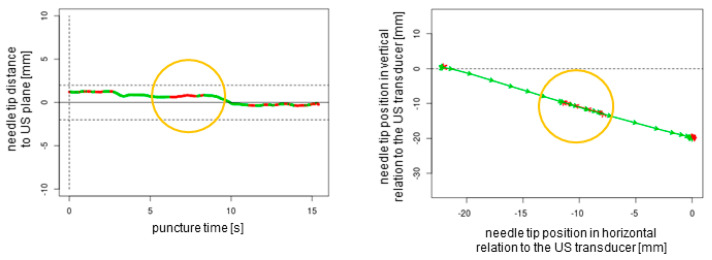
In-plane puncture with readjustment of the needle tip (orange circles). The dotted lines represent the ultrasound transducer planes.

A total of *n* = 240 ex vivo punctures were carried out successfully without any technical failures. In all of the procedures, the needle tip reached the target, confirmed by the endoscopic camera that was inserted into the target tube. For each of the three needles studied (27G 40 mm, 21G 40 mm, and 21G 80 mm), *n* = 80 punctures (*n* = 20 out-of-plane without readjustment, *n* = 20 out-of-plane with readjustment, *n* = 20 in-plane without readjustment, and *n* = 20 in-plane with readjustment) were performed. Each puncture protocol contained 2D plotted analytics of the needle tip movements relative to the plane depending on the time and needle tip direction in horizontal and vertical positions (Figure 4, Figure 5, Figure 6 and Figure 7).

For all punctures, the needle tilt reference was set to 45 degrees, and the initial distance between the needle tip and the center of the target structure was set to 30 mm. For punctures without readjustments, the puncture timer was set to 10 s. For punctures with readjustments, the puncture timer was set to 15 s, and one backward movement of 5 mm was determined (Table 1).

In the statistical comparative analyses, all calculations yielded highly significant values (*p* < 0.001), including (1) an ANOVA for all three needle types across all of the individual measurements; (2) Mann–Whitney U-tests for each needle type between out-of-plane and in-plane punctures (both with and without readjustment); and (3) Mann–Whitney U-tests between conditions with and without readjustment using either puncture technique (out-of-plane or in-plane punctures) for each needle type. Exceptions to these results include datasets where all three needle types showed a relative deviation of 0.0%. The *p*-values were >0.999.

In our ex vivo study model, no system failures occurred. However, system failures can occur in clinical practice under non-optimal examination conditions. Therefore, we conducted ten manual time measurements with a stopwatch following forced system disturbances, showing values of 2.2 s ± 0.3 (2.0, 1.7–2.5).

## 4. Discussion

Needle guidance systems have been proven to enhance both the accuracy and safety of procedures involving sonographically assisted punctures in clinical practice [20,21]. Systems that do not require expensive proprietary needles, instead enabling the use of standard, commercially available needles, are of particular interest [22,23]. The investigated US-NGS shows strong promise to improve both procedural precision and safety, notably without the need for proprietary needles. This presents a clear benefit in clinical environments where cost-effectiveness and operational flexibility are essential. By employing commonly available standard needles, the system integrates seamlessly into routine clinical practices and minimizes reliance on specialized tools.

A primary benefit of this US-NGS is its capability to overlay real-time needle tip location and predicted trajectory onto live ultrasound imagery. This augmented visualization significantly enhances clinicians’ spatial orientation, particularly in out-of-plane procedures where conventional ultrasound provides limited feedback. The addition of software-driven analysis further elevates the system’s effectiveness by offering objective performance assessments through detailed metrics such as needle tilt, insertion depth, and travel distance. These capabilities make the system highly valuable not only in clinical applications but also in educational settings, where structured feedback and skill refinement are crucial.

At our site, the US-NGS under investigation has been successfully used for consecutive fine-needle aspiration cytologies of nearly 250 thyroid nodules without complications [12]. The efficacy of the investigated US-NGS has been demonstrated in several other studies, encompassing a range of simulation and phantom models [13,14,15,16,17]. The system’s capacity to fade in and out the trajectory lines superimposed on the ultrasound image renders it particularly well suited for assessing how successfully trainees are learning. However, these studies did not demonstrate the measurement accuracy and, consequently, the reliability of the US-NGS. The present study was designed to address this critical gap in the existing body of research. Employing state-of-the-art analysis software, this study sought to systematically assess key puncture parameters. These parameters included the movement of the needle tip and tilt, both of which were to be tested under controlled and standardized conditions. The goal was to transfer the clinically experienced gain in precision and security, especially in out-of-plane situations, to an educational setting. In this manner, a customized phantom model-based training workstation was introduced, allowing for the detailed evaluations of several different levels of punctures. Firstly, a top-mounted camera provided visual feedback of the procedure. Secondly, an endoscopic camera located within the tubed target structure showcased the success of the puncture (the phantom was obscure). Thirdly, the movement of the needle tip in relation to the ultrasound probe was documented and analyzed by customized software.

The obtained data could be observed in real time by a trainer positioned at the back of the workstation (not part of the study data); additionally, these data could be recorded for analysis and feedback. The superimposed NGS information can be readily enabled or disabled, a feature that renders the system particularly well suited for educational purposes. Subsequently, the puncture path can be graphically demonstrated to users. This demonstration can elucidate suboptimal puncture paths and corrective movements (Figure 4 and Figure 5). This is particularly helpful as trainee doctors and students may be able to improve their ultrasound puncture skills by reviewing direct visual feedback.

The needles utilized for the thyroid nodule punctures are of a smaller gauge (21–27G) and range in length from 40 to 80 mm. In principle, it is possible that the precision of the NGS’s information may be compromised through bending or interferences within the magnetic field, particularly in the case of thin and elongated needles.

The present phantom study was conducted to ascertain whether the magnet-based US-NGS technique provides reliable data. The target structure was located at a depth of 20 mm, which is a common value for thyroid nodules. However, it should be noted that lesions can be positioned significantly deeper. An analysis was carried out of the movement of the needle tip during 240 standardized punctures using a customized puncture application system (PAS). This study examined three different standard needles: the most commonly utilized needle (21G 40 mm), a thinner needle (27G 40 mm), and a longer needle (21G 80 mm). In all cases, the target was successfully reached, as evidenced by the visual appearance on the endoscopic camera. No system failures were observed.

In a mere two cases (0.8%), the analysis software indicated an additional, unintended, backward movement (one in the in-plane technique and one in the out-of-plane technique). Both instances were meticulously documented for the thinner needle (27G 40 mm). Fornage et al. have emphasized the necessity for potential readjustments during procedures to optimize needle placement [24]. In situations that involve readjustments, the necessity for procedural flexibility to facilitate real-time feedback is particularly pronounced [25,26].

In this context, the investigated US-NGS offers a stable software platform with an intuitive and user-friendly interface, facilitating clinical use after minimal training. The software can be deployed on various hardware configurations, with existing approvals for portable ultrasound systems like the eZono™ 4000 and eZono™ 5000, enabling mobile use in clinical settings. The development of needle guidance software for Android and iOS platforms is already in progress.

The findings of our study indicated only minor discrepancies from the predetermined reference values with regard to the total distance of the needle tip’s movement (comprising forward and backward movements in cases involving readjustments) and the total distance of the needle tip’s reverse movement (only in cases involving readjustments). The software analysis data exhibited a high degree of concordance with the manually adjusted reference values, with relative deviations ranging from −4.2% to +6.7% and a maximum absolute deviation of 1.8 mm (observed in a readjustment in-plane procedure employing the 27G 40 mm needle).

The puncture times indicated by the timer were meticulously followed, with no differences observed between needle types (relative deviations between −2.7% and +3.2%). However, slightly inferior results were obtained for punctures with readjustment. In clinical settings, the importance of brief and uniform puncture times is paramount to minimize tissue trauma and enhance diagnostic yield [27].

Considering the parameters of needle tilt and penetration depth, deviations of less than 1.5% were exhibited by the commonly employed 21G 40 mm needle. These discrepancies were more pronounced with thinner needles (up to a maximum deviation of ~10% for the needle tilt in the in-plane technique) and, to a lesser degree, with longer needles (up to a maximum deviation of ~4% for the needle tilt in the out-of-plane technique). The deviations were attributed to the slight bending of the needle within the phantom tissue [13,28]. Significant deviations can be critical because they impact the biopsy’s accuracy, particularly those related to needle stiffness and operator technique in achieving precise needle positioning [29,30].

The observed deviations highlight the inherent challenges in controlling needle trajectory precisely, influenced by factors such as tissue resistance and needle type [31]. Various studies support these findings, indicating that manual adjustments and patient anatomy significantly impact needle trajectory [32]. All relative deviations stayed below 10%, and the 6 mm target at a depth of 16–23 mm was successfully reached in all cases. Thus, the configuration of the investigated training workstation and the US-NGS utilized is sufficiently precise for the training of novice healthcare professionals. For instance, these parameters can be used to assess the longitudinal outcomes of learning success through repeated applications in educational settings. However, in cases of deeper localized lesions or more complex clinical puncture scenarios, particularly involving thin needles, these deviations can become significant. The most important safety parameters are the penetration depth and needle tilt. The 27G 40 mm needle exhibited maximum deviations of up to 2.6 mm and 6.1 degrees at the respective measurement points. It is imperative to consider these values when operating in close proximity to crucial anatomical structures, such as the trachea or arterial vessels.

In clinical scenarios, a multitude of additional factors must be taken into account. For instance, US-NGS failures may occur in critical situations, such as those resulting from overly steep or shallow angles between the ultrasound and the needle. In the ex vivo measurements conducted, the system stabilized again after approximately two seconds. Therefore, it is imperative to exercise discernment and refrain from uncritically relying on the superimposed needle position information. Instead, the actual visualizations provided by ultrasound imaging should also be considered in clinical punctures.

With regard to the utilized phantom, it is important to note that previous punctures may result in the formation of puncture channels and needle pass artifacts, which can lead to the production of false echo signals and the introduction of visual noise. Artifacts are likely to have a substantial impact on the precision of targeting. However, these phenomena were deemed irrelevant for this study’s design and are thus not the focus of this investigation. In educational environments characterized by a high frequency of repetitive punctures, the utilization of replaceable, interchangeable, and self-made phantom inserts may emerge as a viable solution [33].

The quality of ultrasound devices is continuously improving, enabling more detailed insights into the morphology of even small structures. For instance, it is now feasible to noninvasively assess the microcirculation of testicles using super-resolution ultrasound localization microscopy [34]. These developments will also promote the demand for high-precision ultrasound-assisted puncture techniques. In the future, stable technical systems with precise data transmission, especially with thin needles, will play an important role in the clinical application and training of inexperienced personnel. AI-based training systems hold particular promise to facilitate the establishment of realistic ex vivo practice scenarios [35,36].

### Limitations

It remains unclear whether the discrepancies between the manually set reference values and the data obtained by the customized analysis software are related to inaccurate manual settings or to unstable needle registration. The procedural settings, characterized by the presence of very static puncture paths, do not accurately reflect an in vivo scenario. Clinical circumstances may present a significantly more challenging situation. The optimal puncture angle of 45 degrees can only be utilized in a limited number of cases. The data do not permit the formulation of conclusions regarding the system’s stability at angles that are either acute or steep. However, the objective of this preliminary study was to assess the reliability of the US-NGS at a fundamental level, with the aim of identifying potential registration failures that could potentially compromise clinical interventions.

## 5. Conclusions

The investigation of a magnet-based ultrasound needle guidance system for commercial clinical standard needles revealed the remarkable reliability of the system to determine the position of the needle tip relative to the ultrasound probe. No system failures were observed, and the target was successfully reached in all 240 instances, thereby underscoring the operational robustness of the system. This level of consistency is indicative of the system’s advanced development stage and its suitability for implementation in real-world training and educational environments. However, these findings were observed in an ex vivo phantom model setting under optimal conditions. Therefore, particularly in the context of thin needles, relative deviations of up to 7.5% for penetration depth and 9.6% for needle tilt due to bending must be considered when the system is employed in complex clinical scenarios involving close proximity to critical anatomical structures.

## Figures and Tables

**Table 1:** Performance, safety, and quality parameters for three different needles with or without readjustments in relation to the set reference values.

Parameters	Type	Setup		21G, 40 mm Needle		27G, 40 mm Needle		21G, 80 mm Needle		
		Readjustments	Reference	Measured Value (n = 80) mean ± SD (Median, min–max)	Rel. Deviation [%]	Measured Values (n = 80) mean ± SD (Median, min–max)	Rel. Deviation [%]	Measured Value (n = 80) mean ± SD (Median, min–max)	Rel. Deviation [%]	ANOVA
Puncture time [s]	Out-of- plane	no	10	9.8 ± 0.2 (10.0, 9.4–11.2)	−2.0	10.1 ± 0.3 (10.1, 9.7–10.6)	+1.0	10.0 ± 0.2 (10.0, 9.8–10.3)	±0.0	*p* < 0.001
		yes	15	15.3 ± 0.3 (15.1, 14.4–15.9)	+2.0	14.7 ± 0.5 (15.0, 14.4–15.5)	−2.0	15.5 ± 0.6 (15.1, 14.8–16.0)	+3.2	*p* < 0.001
	In- plane	no	10	10.1 ± 0.2 (10.0, 9.8–10.4)	+1.0	9.8 ± 0.3 (9.8, 9.4–10.5)	−2.0	9.7 ± 0.4 (10.0, 9.4–10.3)	−3.1	*p* < 0.001
		yes	15	14.6 ± 0.5 (14.9, 14.1–15.4)	−2.7	15.3 ± 0.5 (15.2, 14.6–16.2)	−2.0	15.4 ± 0.5 (15.1, 14.7–15.6)	+2.6	*p* < 0.001
Total distance of needle tip movement [mm]	Out-of- plane	no	30	30.3 ± 0.3 (30.1, 29.6–30.7)	+1.0	29.0 ± 1.0 (29.8, 28.6–30.5)	−3.4	29.3 ± 0.8 (29.8, 28.9–30.5)	−2.4	*p* < 0.001
		yes	40	39.8 ± 0.3 (39.9, 39.5–40.8)	−0.5	40.9 ± 0.9 (40.1, 39.5–41.7)	+2.2	40.2 ± 0.3 (40.0, 39.7–40.8)	+0.5	*p* < 0.001
	In- plane	no	30	29.8 ± 0.3 (30.0, 29.6–30.5)	−0.3	30.8 ± 0.9 (29.9, 29.3–31.2)	+2.6	30.6 ± 0.6 (29.9, 29.3–30.9)	+2.0	*p* < 0.001
		yes	40	40.3 ± 0.4 (40.1, 39.6–40.9)	+0.7	38.5 ± 1.5 (39.6, 38.2–40.2)	+3.9	39.0 ± 1.0 (39.8, 38.6–40.4)	+2.6	*p* < 0.001
Maximum penetration depth of needle tip [mm]	Out-of- plane	no	20	20.0 ± 0.7 (19.9, 18.4–21.3)	±0.0	20.5 ± 0.5 (20.5, 19.3–21.1)	+2.5	19.7 ± 0.3 (19.9, 18.9–20.7)	−1.5	*p* < 0.001
		yes	20	19.8 ± 0.3 (20.0, 19.5–20.3)	−1.0	18.5 ± 0.1 (18.5, 18.3–18.7)	−7.5	20.2 ± 0.4 (19.9, 19.8–20.9)	+1.0	*p* < 0.001
	In- plane	no	20	20.1 ± 0.2 (20.1, 19.8–20.5)	+0.5	20.8 ± 0.9 (21.0, 19.7–22.4)	+4.0	20.3 ± 0.4 (20.1; 19.6–21.0)	+1.5	*p* < 0.001
		yes	20	20.2 ± 0.3 (20.2, 19.7–20.6)	+1.0	20.9 ± 0.8 (21.0, 20.4–22.6)	+4.5	19.7 ± 0.6 (20.1, 19.0–21.0)	−1.5	*p* < 0.001
Needle tilt [degrees]	Out-of- plane	no	45	45.5 ± 0.7 (45.1, 44.0–45.8)	+1.1	48.1 ± 2.4 (46.0, 43.1–50.8)	+6.4	44.0 ± 1.1 (44.3, 43.8–47.9)	−2.2	*p* < 0.001
		yes	45	44.5 ± 1.0 (44.9, 43.9–46.2)	−1.1	48.4 ± 2.1 (46.0, 44.8–49.3)	+7.0	43.3 ± 1.6 (44.8, 42.9–48.7)	−3.9	*p* < 0.001
	In- plane	no	45	44.8 ± 0.4 (45.0, 44.0–45.6)	−0.4	42.3 ± 1.8 (42.5, 40.0–46.0)	−6.4	46.0 ± 1.2 (45.0, 44.0–47.1)	+2.2	*p* < 0.001
		yes	45	45.7 ± 0.9 (45.3, 44.1–47.2)	+1.3	49.8 ± 2.6 (48.3, 44.8–51.1)	+9.6	43.8 ± 1.5 (44.7, 43.0–47.4)	−2.7	*p* < 0.001
Number of trajectory readjustments [n]	Out-of- plane	no	0	0.0 ± 0.0 (0.0, 0.0–0.0)	±0.0	0.1 ± 0.2 (0.0, 0.0–1.0)	±0.0	0.0 ± 0.0 (0.0, 0.0–0.0)	±0.0	*p* > 0.999
		yes	1	1.0 ± 0.0 (1.0, 1.0–1.0)	±0.0	1.0 ± 0.0 (1.0, 1.0–1.0)	±0.0	1.0 ± 0.0 (1.0, 1.0–1.0)	±0.0	*p* > 0.999
	In- plane	no	0	0.0 ± 0.0 (0.0, 0.0–0.0)	±0.0	0.0 ± 0.0 (0.0, 0.0–0.0)	±0.0	0.0 ± 0.0 (0.0, 0.0–0.0)	±0.0	*p* > 0.999
		yes	1	1.0 ± 0.0 (1.0, 1.0–1.0)	±0.0	1.1 ± 0.3 (1.0, 1.0–2.0)	+10.0	1.0 ± 0.0 (1.0, 1.0–1.0)	±0.0	*p* > 0.999
Total distance of reverse needle tip movement [mm]	Out-of- plane	no	0	0.0 ± 0.0 (0.0, 0.0–0.0)	±0.0	0.1 ± 0.1 (0.0, 0.0–0.3)	±0.0	0.0 ± 0.0 (0.0, 0.0–0.0)	±0.0	*p* > 0.999
		yes	5	5.1 ± 0.2 (5.0, 4.8–5.3)	+2.0	4.8 ± 0.3 (5.0, 4.3–5.4)	−4.2	4.9 ± 0.2 (5.0, 4.7–5.2)	−2.0	*p* < 0.001
	In- plane	no	0	0.0 ± 0.0 (0.0, 0.0–0.0)	**±0.0**	0.0 ± 0.0 (0.0, 0.0–0.0)	**±0.0**	0.0 ± 0.0 (0.0, 0.0–0.0)	**±0.0**	*p* > 0.999
		yes	5	4.9 ± 0.1 (5.0, 4.9–5.2)	**−2.0**	5.3 ± 0.4 (5.0, 4.7–5.6)	**+6.7**	4.8 ± 0.3 (5.0, 4.5–5.3)	**+4.2**	*p* < 0.001

## Data Availability

The original contributions presented in this study are included in the article and Supplementary Materials. Further inquiries can be directed to the corresponding author.

## Supplementary Materials

The following supporting information can be downloaded at: https://www.mdpi.com/article/10.3390/s25134102/s1, Video S1: The image displays a 10-s sequence of an out-of-plane measurement on the phantom. The left image displays the screen of the eZono interface, accompanied by a bird’s-eye perspective of the environment and the endoscope camera view on the right.
